# 1283. High Dose Minocycline for the Treatment of Acinetobacter Infections

**DOI:** 10.1093/ofid/ofab466.1475

**Published:** 2021-12-04

**Authors:** Shelbye R Herbin, Amina Ammar, Ryan Gumbleton, Jing Zhao, Shannon Olson, Stephanie M Smith, Marco R Scipione

**Affiliations:** 1 Detroit Receiving Hospital, Ferndale, Michigan; 2 Detroit Medical Center, Dearborn, Michigan; 3 Detroit Medical Center-Harper Hospital, Detroit, Michigan; 4 Sinai-Grace Hospital Detroit Medical Center, Detroit, Michigan; 5 Children’s Hospital of Michigan, Detroit, Michigan

## Abstract

**Background:**

Acinetobacter (ACB) infections are difficult to treat due to increasing drug resistance. The aim of this study is to evaluate the effectiveness of high-dose (HD) minocycline (MIN) 200mg q12h for the treatment of ACB infections

**Methods:**

This is a retrospective study of pts with ACB from 1/1/19 to 10/31/20. Exclusions included non-susceptibility to MIN, age < 18 yrs, hospice care w/in 72 hrs of culture, or urine source. Use of HD MIN (IV or PO), was compared to alternative treatment (AT). Clinical and micro success at the end of therapy (EOT) was evaluated. Clinical success was elimination or improvement in signs and symptoms of infection. Micro success was eradication of ACB from the same site of infection at EOT. Length of stay (LOS), 30-day readmission (with or without ACB), isolation of a MIN non-susceptible ACB within 30 days of EOT, and adverse events related to MIN, were evaluated.

**Results:**

A total of 320 pts were screened with the most common exclusion being MIN non-susceptible isolate. Of the 204 pts included, 38 received HD MIN and 166 received AT. The most common were cefepime (53/166) and meropenem (36/166). Median age (IQR) was 41 (30-50) yrs for HD MIN vs. 40 (27-48) yrs for AT. Both groups were mostly male (HD MIN: 63% vs. AT: 60%) and respiratory was the most common site (HD MIN: 61% vs. AT: 60%). HD MIN group had 74% of cultures that were polymicrobial vs. 86% in AT group. Lack of clinical response at the EOT occurred in 42% of pts on HD MIN and 37% on AT. Infection related mortality occurred in 24% on HD MIN vs. 15% on AT. In HD MIN group, 16 pts had a repeat culture from the same site after treatment with 25% still positive for ACB. In the AT group 61 pts had a repeat culture and 28% were positive for ACB. Duration of treatment (9 [6-11] days vs. 10 [2-18] days) and LOS (16 [18.25-26.75] vs. 29 [14-49]) were shorter in the HD MIN group. There was only one adverse event reported with the use of HD MIN

**Conclusion:**

Pts in the HD MIN group had shorter treatment duration and a shorter LOS. Pts who received HD MIN had a lower rate of clinical cure and a higher mortality rate compared to pts in the AT group. Reasons for this difference will need to be investigated further. HD MIN should be reserved for pts who are unable to tolerate other treatment options or who have an ACB resistant to other treatment options.

**Disclosures:**

**All Authors**: No reported disclosures

